# Caring for the carers: safeguarding oncologists’ mental health in the time of COVID-19

**DOI:** 10.3332/ecancer.2020.1057

**Published:** 2020-06-15

**Authors:** Louis Mervyn B Leones, Carlo Miguel P Berba, Alfredo V Chua, Jennifer Sandoval-Tan

**Affiliations:** Division of Medical Oncology, Department of Medicine, University of the Philippines—Philippine General Hospital, Manila, Philippines; a http://orcid.org/0000-0002-7927-2441; b http://orcid.org/0000-0002-0664-4304; c http://orcid.org/0000-0002-0895-8139

**Keywords:** mental health, oncologist, COVID-19

## Abstract

Taking care of patients with chronic, terminal diseases presents unique challenges to the mental health of medical oncologists. The current coronavirus disease 2019 (COVID-19) pandemic has exacerbated these mental health risks brought about by isolation and exhaustion. Delegated to be a national COVID-19 referral centre, the University of the Philippines—Philippine General Hospital faced many challenges, including the increased workload in a perilous and anxiety-inducing national crisis which placed the entire healthcare team in an unprecedented situation. To adapt to these challenges, the Division of Medical Oncology employed the following measures to safeguard the mental health of its faculty and fellows: 1) use of psychological support materials; 2) initiation of a psychological intervention programme and 3) establishment of peer support programmes. Caring for the carers through evidence-based interventions ensures the delivery of quality care to our cancer patients despite the challenges during these trying times.

## Introduction

 The practice of oncology is often seen as heavy for the psyche. Daily encounters with patients who have chronic, terminal diseases present unique challenges to mental health, possibly leading to burnout [1]. This may be from the burden of making life-and-death decisions and unsatisfactory work-life balance in the face of an often limited ability to significantly prolong the life of most patients. When these issues go unaddressed, suicide risk is increased [1]. However, studies focusing on how to address these issues particularly among oncologists are limited [1].

 The coronavirus disease 2019 (COVID-19) pandemic has exacerbated the mental health risks of physicians. As seen in the severe acute respiratory distress syndrome (SARS) experience, psychological distress, fear, anxiety and post-traumatic stress symptoms have profound impacts [2]. On top of concerns of risk of infection, physicians also face additional stressors such as isolation and exhaustion [3]. They were noted to have a higher risk of depression, anxiety, insomnia and distress [4]. These effects are related to the physicians’ department and occupation [5]. How these specifically apply to oncologists, however, is yet to be known.

Starting March 2020, the University of the Philippines—Philippine General Hospital (UP-PGH), the 1,500-bed national university hospital, has been delegated to be a national COVID-19 referral centre. Several changes ensued, including limiting elective admissions and a near-total temporary closure of outpatient services. The Cancer Institute, however, remained operational while working on a skeleton workforce. Because of the conversion of various areas into new COVID-19 wards, medical personnel were transferred temporarily to augment manpower in these areas. This increased workload put the entire team in an unprecedented situation.

To adapt to the situation, the Division of Medical Oncology employed the following measures to safeguard the mental health of its faculty and trainees:

## Use of psychological support materials

Support materials were provided both online and offline. The university’s information office has published *Healthscape*, a weekly COVID-19 newsletter. In its fourth issue, it outlined the hospital’s endeavors including projects like *Resiliency Wall*, a closed group page for information dissemination and discussion of COVID-19-related issues; psychosocial care posters with infographics; and *Heroes’ Heroes,* an initiative that aims to collect thank you cards and words of encouragement for health workers [6]. Recommendations from the Philippine Council for Mental Health are also available in the hospital’s social media page.

## Initiation of a psychological intervention programme

Recognising the role of therapist-driven sessions [7], the division collaborated with the Department of Psychiatry for a psychological intervention programme. An online survey was conducted to identify self-perceived mental health status and issues. Twelve of the 14 fellows answered standardised questionnaires on anxiety, depression and burnout. Participants reported that the seriousness of the disease and the current situation coupled with the responsibility of taking care of cancer patients contributed to the anxiety felt, especially when on duty at the COVID-19 areas. Internal strengths and resources identified that could help were the good support system between the faculty and trainees, and guidance on how to manage issues arising from this crisis. One major limitation is the limited access to funding resources for cancer patients because most funding agencies, both government and private, directed their funds to COVID-19-related interventions.

A psychiatrist then conducted regular group sessions via an online meeting platform. Digital technologies provide a range of mental health interventions [8]. Separate meetings were held for the first-year fellows, second-year fellows and faculty to ensure an avenue for the safe discussion of possible issues and challenges that they were facing. Processing of their psychosocial reactions was done, and coping strategies were explored. Their mental readiness to continue with the division’s activities was also assessed.

Sixteen of the 22 oncologists participated. The psychosocial reactions expressed were similar among the groups. Anxiety and fear were predominant themes, consistent with the results of the survey. They expressed fear for their safety, specifically about the possibility of contracting the disease, their families’ and co-workers’ safety, and the well-being of their patients. In particular, the new first-year fellows expressed doubts about their capacity to perform their tasks satisfactorily, especially at the COVID-19 areas, because of their unfamiliarity with the new set-up.

An important internal factor which helped with coping was their strong sense of responsibility as doctors and members of the community. This gave meaning to their role despite the uncertainties of the situation. Their strong sense of group spirit with the mindset that the crisis is a shared burden also helped. The concrete and visible safety efforts implemented by the hospital, help from various organisations, and consistent efforts of the division to check on each other’s well-being were some external factors which allayed anxiety. Other coping mechanisms include staying connected with family and friends, being productive and engaging in enjoyable activities.

With these findings, the Department of Psychiatry deemed the oncologists to have appropriate psychosocial reactions. Emotional and cognitive coping mechanisms were employed and were adaptive to the stresses of the pandemic. Thus, they were assessed to be mentally ready to continue with the divisions’ activities.

## Establishment of peer support programme

Peer support programmes were in place. Colleagues and immediate supervisors provided emotional and psychological support [9]. At the start of the training programme, faculty members were assigned trainees whom they would mentor. During the pandemic, this coupling was utilized as a means of top–down emotional support. Mentors and mentees would have weekly virtual meetings, where the faculty could process pertinent events during the week and identify any issues among the trainees. These meetings provided the oncologists a structured psychological safety net and a venue for the discussion of clinical issues that may arise during the delivery of cancer care.

Colleagues also contributed to the supportive environment by participating in a buddy system, where each junior fellow was paired with a senior. They would often be assigned together during hospital duties and assignments. Although less structured than a mentor-mentee system, this provided immediate support in a more collegial environment.

## Evaluation

The long-term effects of these ongoing interventions are still to be evaluated. Periodic evaluation by the Department of Psychiatry in the form of regular scheduled meetings and answering of standardised questionnaires on anxiety, depression and burnout is continued. Should the nationwide pandemic start to ebb, the terminal component of this support programme will be evaluated by group and individual interviews, as well as monitoring trends in anxiety, depression and burnout.

## Conclusions

The pressing need to ensure that the mental health needs of medical oncologists are met in an unprecedented time like this cannot be overemphasised. Although challenging, mitigating the negative psychological impacts of being an oncologist is vital in winning against COVID-19. Caring for the carers through evidence-based interventions ensures the delivery of quality care to our cancer patients.

## Conflicts of interest

The authors declare that they have no conflicts of interest.

## Funding statement

No funding was received for this work.
